# Complete Laparoscopic Extirpation of a Giant Ovarian Cyst in an Adolescent

**DOI:** 10.1155/2017/7632989

**Published:** 2017-08-03

**Authors:** Saeed Baradwan, Feras Sendy, Sameer Sendy

**Affiliations:** Department of Obstetrics and Gynecology, King Fahad Medical City, Riyadh, Saudi Arabia

## Abstract

The giant ovarian serous cystadenoma is a rare finding and often benign. The use of the laparoscopic approach versus open approach for the management of huge ovarian cysts is controversial. We report a case of a 27-year-old woman with a history of increasing abdominal girth over a period of two years along with radiological investigations revealed a large tumor arising from the right ovary treated by complete laparoscopic extirpation of a giant ovarian cyst. The complete laparoscopic approach for huge cyst is a feasible treatment when having a normal tumor marker profile and benign imaging appearance. In addition to the advantages of laparoscopic surgery, it is less invasive, with perfect cosmetic outcome and shorter hospital stay, which are particularly important for young women.

## 1. Introduction

The giant ovarian cyst is rarely found nowadays because of the improved imaging modalities and minimally invasive approaches so the diagnosis is made at an earlier stage. Serous cystadenomas are the most common types of epithelial neoplasms encountered with benign cystadenoma about 75% [1]. Management of ovarian cysts depends on the patient's age, the size of the cyst, and its histopathological nature which is conservative surgery as ovarian cystectomy and salpingo-oophorectomy is adequate for benign lesions [2].

We report a case of a woman with a history of increasing abdominal girth over a period of two years along with radiological investigations revealed a large tumor arising from the right ovary treated by complete laparoscopic extirpation of a giant ovarian cyst.

## 2. Case Report

A 27-year-old single Saudi woman presented with her parents on September 2016 at the gynecology outpatient clinic of the King Fahad Medical city, Saudi Arabia, with a gradually abdominal distension and discomfort over two years. The swelling was accompanied by vague abdominal pain and breathlessness which worsened on recumbency leading to decreased sleep. She also complained of constipation and poor appetite. There was no history of vomiting or other gastrointestinal symptoms, urinary symptoms, colicky pain, and fainting attacks. She had no previous history of any illnesses, allergies, or operations. She denied the use of any medications. There was no family history of malignancies. Her menarche commenced at the age of 11 years with subsequent regular cycles.

Her body weight was 71 kg, her height was 157 cm, and her BMI was 28.8. On physical examination, she was afebrile with a pulse rate of 79 b/min, blood pressure of 120/73 mm Hg, and respiratory rate of 20 cycles/min. There was no jaundice, edema, or lymphadenopathy. Secondary sexual characteristics were evident. Abdominal examination revealed abdomen was distended with large ill-defined pelvic-abdominal cystic mass extending from pubis to epigastrium with an abdominal girth of 106 cm with a dull note on percussion, without tenderness and the presence of dermal striae. Intestinal sounds were normal. The external genital examination was normal.

**Figure 1:**
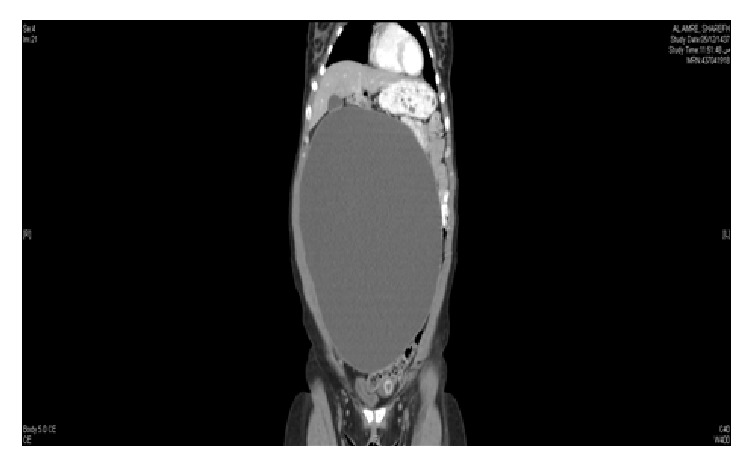
Abdominopelvic computed tomography (CT) showing a large well-defined homogeneously cystic lesion originates from the right ovary measuring 15.6 × 26.3 × 31.7 cm.

Plain radiograph of the chest (P-A view) was within normal limits. Transabdominal ultrasonography verified a large pelvic cystic mass about 29 × 18 cm with no evidence of solid components or septations. The uterus was normal and endometrial thickness was 10 mm. CA-125 was 25.3 IU/ml and another tumor marker was within the normal ranges. Abdominopelvic computerized tomography (CT) findings were consistent with a large well-defined homogeneously cystic lesion originating from the right ovary measuring 15.6 × 26.3 × 31.7 cm in the AP, transverse, and craniocaudal dimensions, respectively (Figure 1). Most likely this represents ovarian cystadenoma, with no abdominopelvic metastases or lymphadenopathy. Our patient was counseled and signed informed consent for laparoscopic ovarian cystectomy, possible oophorectomy, and laparotomy if needed.

**Figure 2:**
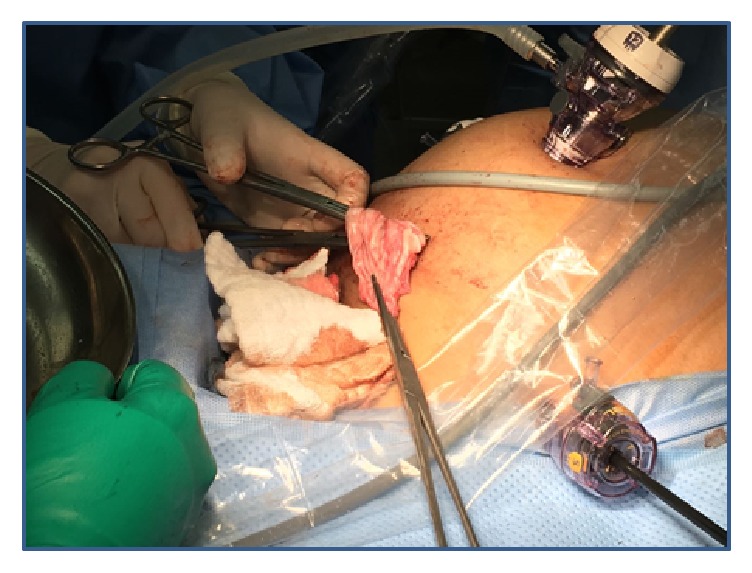
The cyst wall components removed through suprapubic opening.

**Figure 3:**
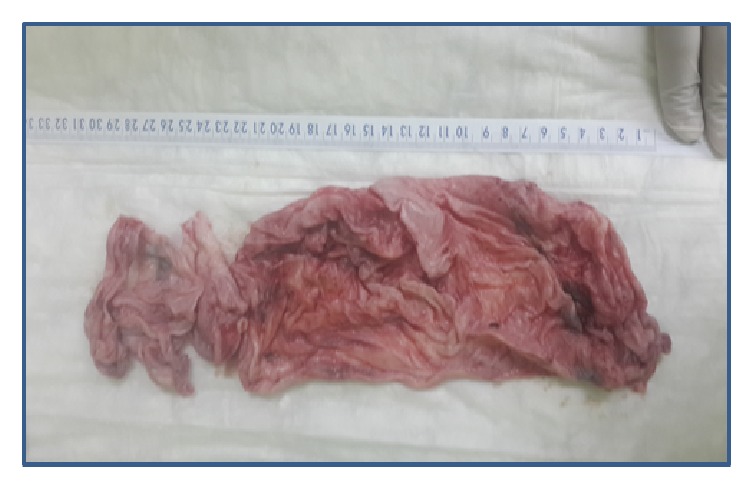
Total excision of the cyst measured around 31 cm.

Intraoperatively, the patient was in a supine position, prepped and draped in the usual sterile fashion. The bladder had been drained with a Foley catheter that was placed in the bladder. Attention was then turned to the abdomen where an approximately 10 mm skin incision was made in the umbilical fold. Entry into the peritoneum was done using the Hasson technique. The 10 mm trocar was placed through the umbilical incision. The abdominal cavity was visualized with huge cyst arising from the right ovary which was seen, extending up to the level of the xiphoid process, and the liver was not visualized due to the huge cyst. Peritoneal wash was sent for cytology. The 10 mm second trocar was applied in the suprapubic catheter with direct opening in the cyst and being drained around 10,050 ml. However, with suction, a large globular piece of the cyst was obtained that appeared to be coagulated blood which was sent for cytology. Two trocars 5 mm were inserted in the lower quadrant bilaterally. The cyst wall was identified and was removed using blunt dissection with countertraction. The ovarian bed was then irrigated and dried. There was slight oozing noted near the edge of the ovary, with hemostasis obtained using electrocautery. The Endo Catch bag was placed through the 10 mm port (suprapubic) and the cyst wall components, and the right ovarian cyst was placed in the Endo Catch bag and removed through the 10 mm incision which was sent for histopathology (Figure 2). After decompression of the ovarian cyst, the liver and the gallbladder were seen with no abnormality. The uterus and the left ovary were looking normal. Lymph nodes were not enlarged. The pelvis was copiously irrigated and dried. Hemostasis was assured. Operative time was 155 minutes. Estimated blood loss was minimal. The patient tolerated the procedure well. Total excised cyst measured 31 cm in size and 550 grams by weight (Figure 3). Total volume of the cyst was calculated to approximately 11 kg. The postoperative period was uneventful.

She was discharged on the 2nd postoperative day. The patient was advised to follow up after 4 weeks. Patient weighed 60 kg with an abdominal girth of 80 cm on day 28 after operation. Cytological examinations were negative for malignancy. Histopathological examination revealed serous cystadenoma of the ovary. She recovered completely from her surgery and has gone back to her normal daily activity.

## 3. Discussion

Small ovarian cysts are usually asymptomatic and found incidentally clinically or on ultrasound. They may sometimes cause pain or discomfort. The benign neoplastic cysts are most frequently endometrial or chocolate cyst or simple cyst. The most frequent complications of benign ovarian cysts are torsion, hemorrhage, and rupture [3]. There are many differential diagnoses with ovarian cysts like functional cysts, omental cysts, mesenteric cysts, urinary retention, bladder diverticulum, hydronephrosis, cystic lymphangiomas, gastrointestinal duplication cysts, and large uterine tumors [4].

Serous cystadenomas are the most common types of epithelial neoplasms encountered with benign cystadenoma about 75% and mucinous cystadenomas were 25%. Serous or mucinous cystadenomas of the ovary, benign or malignant, are rare in children. They arise from Mullerian germinal epithelium [1]. Giant ovarian serous cystadenomas of such a huge size are a rare finding and are often benign. The most remarkable descriptions of large ovarian cysts are those of Spohn, who in 1922 reported that one weighed 148.6 kg (328 lb), and Symmonds, who in 1963 reported encountering one that weighed 79.4 kg (175 lb) [5]. Furthermore, Zamora et al. (1992) reported 72 kg ovarian cyst whereas Farinetti et al. reported excision of 23 kg ovarian cyst [6, 7].

In laparoscopic surgery with the availability of modern advanced techniques and expertise in minimally invasive surgery, laparoscopic excision is preferred in the management of giant ovarian cyst extending up to the level of the umbilicus mainly due to its least invasiveness, better cosmesis, and shorter hospital stay, but only a few cases have been reported [8]. All reported techniques include decompression of the cyst, facilitate manipulation of the cyst and ovary, and prevent perforation and spillage. One case of giant ovarian cyst 21 cm underwent cyst drainage with paracentesis during diagnostic laparoscopy, followed by laparoscopic removal of the cyst and left adnexa [9]. Other giant ovarian cyst 22 cm was managed with prelaparoscopy cyst drainage with a suprapubic catheter followed by laparoscopic cystectomy [10]. To our knowledge, there are no guidelines in the literature regarding the maximal size of cyst which can be considered for laparoscopic surgery. With proper patient selection, laparoscopic surgery can be safely applied in a select group of patients with large, benign ovarian cyst [10]. In young women, one of the main goals is to preserve the reproductive and hormonal functions of the ovaries and prevent recurrence.

## 4. Conclusion

The complete laparoscopic extirpation of a giant ovarian cyst is a feasible treatment when having a normal tumor marker profile and benign imaging appearance. In addition to the advantages of laparoscopic surgery, it is less invasive, with perfect cosmetic outcome and shorter hospital stay, which are particularly important for young women.
